# Computational genetic neuroanatomy of the developing mouse brain: dimensionality reduction, visualization, and clustering

**DOI:** 10.1186/1471-2105-14-222

**Published:** 2013-07-11

**Authors:** Shuiwang Ji

**Affiliations:** 1 Department of Computer Science, Old Dominion University, 4700 Elkhorn Avenue, Suite 3300, Norfolk, VA 23529-0162, USA

## Abstract

**Background:**

The structured organization of cells in the brain plays a key role in its functional efficiency. This delicate organization is the consequence of unique molecular identity of each cell gradually established by precise spatiotemporal gene expression control during development. Currently, studies on the molecular-structural association are beginning to reveal how the spatiotemporal gene expression patterns are related to cellular differentiation and structural development.

**Results:**

In this article, we aim at a global, data-driven study of the relationship between gene expressions and neuroanatomy in the developing mouse brain. To enable visual explorations of the high-dimensional data, we map the *in situ* hybridization gene expression data to a two-dimensional space by preserving both the global and the local structures. Our results show that the developing brain anatomy is largely preserved in the reduced gene expression space. To provide a quantitative analysis, we cluster the reduced data into groups and measure the consistency with neuroanatomy at multiple levels. Our results show that the clusters in the low-dimensional space are more consistent with neuroanatomy than those in the original space.

**Conclusions:**

Gene expression patterns and developing brain anatomy are closely related. Dimensionality reduction and visual exploration facilitate the study of this relationship.

## Background

The brain consists of an enormous number of cells organized into structures [1,2]. The structured organization of cells is the key to the functional efficiency of the brain [3-6]. Hence, a natural first step toward understanding the brain function would be to address basic research questions at the structure level. How cells are organized into structures [7,8]? What are the functions of structures [9]? How the structures are connected to each other [10,11]? However, a fundamental difficulty of understanding brain functions at the structure level lies in that there is no universally agreed division of cells into structures [12].

From a developmental perspective, the delicate organization of brain into structures is the consequence of stringent spatiotemporal patterning controlled by the molecular signals during development. In this process, cells at different spatial locations read different morphogenetic positional signals produced by the graded distribution of signaling molecules. These signals control the expression of a relatively small set of transcription factors, which in turn regulate the expression of a larger number of genes. This sequential cascade of expression control ultimately leads to cell differentiation and the emergence of connections and functional properties [13]. The discovery that certain marker genes are expressed in regionally restricted patterns in the developing brain has either led to the introduction of new structural boundaries or made it possible to re-define existing boundaries at a higher resolution [14]. Currently, studies on the molecular-structural associations are beginning to reveal how the spatiotemporal gene expression patterns are related to cellular differentiation and structural development [15-18].

In this article, we study the relationship between brain anatomy and spatiotemporal gene expression patterns in the developing mouse brain. This global study of developing neuroanatomy is made possible by the high-resolution, three-dimensional (3-D) gene expression patterns provided by the Allen Brain Atlas (ABA) [19-22]. As part of the ABA, the Allen Developing Mouse Brain Atlas provides spatiotemporal *in situ* hybridization (ISH) gene expression pattern images across four embryonic and three postnatal developmental ages [21,22], yielding effectively a four-dimensional brain atlas. To establish a common coordinate framework for analyzing the ISH data, the ISH image series are aligned to the Allen Developing Mouse Brain Reference Atlas. This enables the global, computational study of the spatiotemporal gene expression patterns of many genes and comparison of the results with developmental anatomy.

To enable visual explorations of the gene expression patterns and correlate the results with classically defined neuroanatomy, we first map the high-dimensional, voxel-level gene expression data to low-dimensional space in which data visualization can be readily achieved. Numerous multivariate analysis methods can be used for this purpose. However, traditional methods either retain the global structures or the local structures in computing the mapping, producing results that are not satisfactory. To preserve both the local and the global structures in the spatial gene expression space, we employ a recent method known as the *t*-distributed stochastic neighbor embedding (t-SNE) [23] for mapping the high-dimensional data. This method is able to capture the local similarities in the high-dimensional space, while retaining the global structures as much as possible.

We map the high-dimensional gene expression data to 2-D space using t-SNE and visualize the reduced data at multiple levels of the Allen Developing Mouse Brain Reference Atlas ontology, which was created based on the “prosomeric model” [24-26]. This models proposes that the neural tube is divided into grid-like pattern of longitudinal and transverse regions. Our results show that the brain anatomy is largely preserved in the low-dimensional gene expression space at multiple levels. To provide a quantitative comparison of the relationship between gene expression patterns and neuroanatomy, we cluster the brain voxels into groups based on gene expression data in the original high-dimensional space and in the dimensionality-reduced space. Our results show that the clustering results in the low-dimensional space are more consistent with developmental anatomy than those in the original high-dimensional space.

## Methods

### Allen developing mouse brain atlas

The Allen Developing Mouse Brain Atlas (the Atlas) contains spatiotemporal *in situ* hybridization (ISH) gene expression data across multiple stages of mouse brain development [19,21]. The primary data consist of 3-D, cellular resolution ISH expression patterns of approximately 2000 genes in sagittal plane across four embryonic (E11.5, E13.5, E15.5, and E18.5) and three early postnatal ages (P4, P14, and P28). The ISH image series are processed by an informatics pipeline at the Allen Institute for Brain Science [27]. To establish a common coordinate framework for analyzing the ISH data, the ISH image series are aligned to the Reference Atlas in 3-D space. After the ISH image series are mapped to the reference space, a gridding module is applied to divide the 3-D reference space into regular grid. The resolution of the data grids varies with age and are shown in Table 1 along with the sizes of each dimension. For each grid voxel, an expression energy value is extracted. All downstream analysis functions provided by the Allen Brain Atlas, such as the anatomic search, gene search, and temporal search, are based on the expression energy. Our analysis in this work is also based on the grid-level expression energy.

**Table 1:** The sizes of the 3-D grid data arrays at seven developmental ages

**Age**	**Grid resolution**	**x-dimension size**	**y-dimension size**	**z-dimension size**
	**(in micron)**	**(anterior-to-posterior)**	**(superior-to-inferior)**	**(left-to-right)**
E11.5	80	70	75	40
E13.5	100	89	109	69
E15.5	120	94	132	65
E18.5	140	67	43	40
P4	160	77	43	50
P14	200	68	40	50
P28	200	73	41	53

The 3-D reference space is in PIR orientation where x axis corresponds to anterior-to-posterior, y axis corresponds to superior-to-inferior, and z axis corresponds to left-to-right.

**Figure 1:**
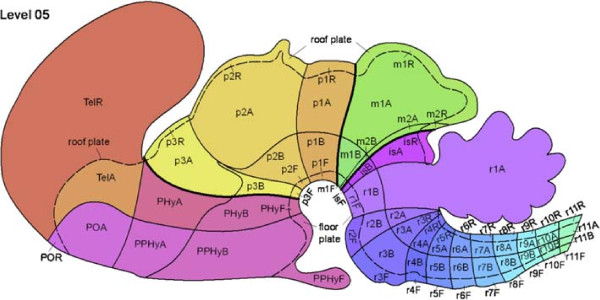
**Illustration of the prosomeric model on which the Allen Developing Mouse Brain Reference Atlas is based.** This figure shows the grid-like pattern corresponding to the Reference Atlas ontology levels up to 5. The figure is reproduced from Figure 3 in [28] with permission.

**Figure 2:**
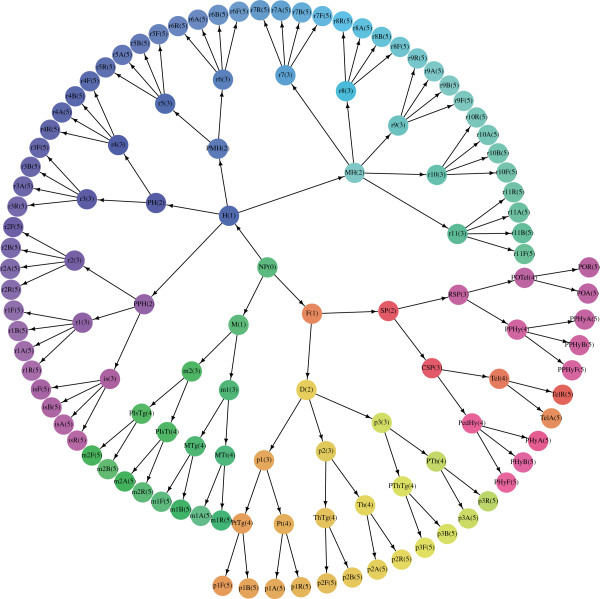
**The Allen Developing Mouse Brain Reference Atlas ontology hierarchy through level 5.** Each ontological term corresponds to a node in the hierarchy, labeled by the abbreviation followed by the level number inside a parenthesis. The nodes are color-coded as in the original atlas in Figure 1. The transverse segments lie at level 3, and they are combined with the longitudinal zones at level 5 to generate the grid-like pattern. We up-propagate the voxel annotations to levels 1, 3, and 5, respectively, in our experiments in order to study the gene expressions in the grid-like longitudinal and transversal domains.

**Figure 3:**
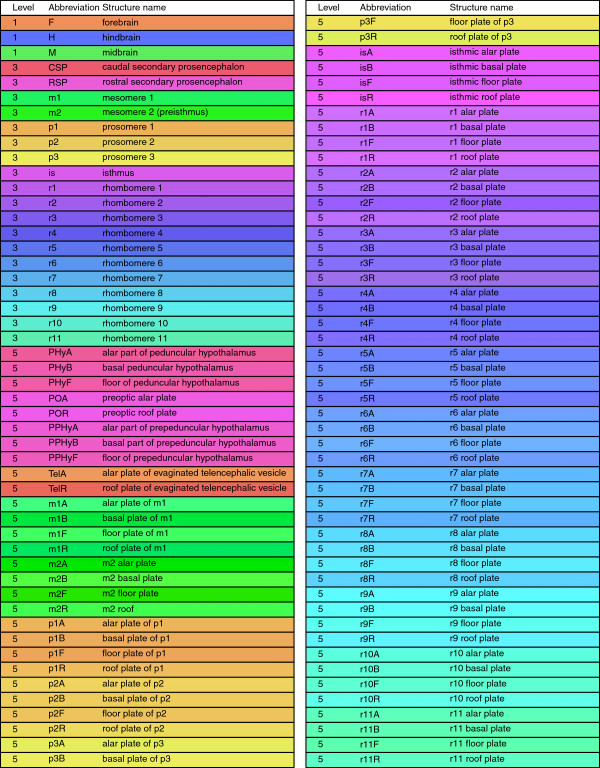
**The list of terms in the Allen Developing Mouse Brain Reference Atlas ontology levels 1, 3, and 5.** We show the level, abbreviation, and structure name of each brain structure in the ontology in a box that is colored as in the Reference Atlas.

The Reference Atlas ontology was created based on the prosomeric model, which proposes that the developing brain is divided along the transversal and longitudinal boundaries, giving rise to a grid-like pattern (Figure 1). The ontology was designed to capture the progressive development and regionalization of the nervous system. An ontological term at each level has multiple child terms at the next level, reflecting the subdivision of the corresponding structure into multiple substructures. By this construction, the Reference Atlas ontology forms a 13-level hierarchy in which the root corresponds to the undivided neural plate. The ontology was colorized so that anatomically and developmentally related structures are coded with similar colors. The ontology from Level 0 to Level 5 is shown in Figures 2 and 3.

### Dimensionality reduction and visualization

Dimensionality reduction is the procedure of mapping high-dimensional data points to low-dimensional space by optimizing certain criterion. Such techniques facilitate visual exploration of the high-dimensional data when they are mapped to 2-D or 3-D space. Traditional techniques for dimensionality reduction include linear method such as principal component analysis (PCA), multidimensional scaling (MDS), and nonlinear approaches such as local linear embedding (LLE) [29,30]. These techniques either capture the global structure of the original data or try to retain the local structure within the neighborhood of each data point.

In order to capture both the local structure and the global structure such as the presence of clusters, a class of methods, known as the stochastic neighbor embedding (SNE), have been developed [31]. To simplify the optimization and overcome the so-called “crowding problem”, SNE is extended to *t*-distributed SNE (t-SNE) in [23]. Given *n* high-dimensional data points ${\left\{x_{i}\right\}}_{i=1}^{n}$ where $x_{i}\in {\mathbb{R}}^{d}$, t-SNE computes *n* low-dimensional data points ${\left\{y_{i}\right\}}_{i=1}^{n}$, known as map points, by trying to preserve the pairwise similarities in the high-dimensional space. To this end, t-SNE computes an *n* × *n* similarity matrix in both the original data space and in the low-dimensional space. The similarity matrix in the high-dimensional space is obtained based on symmetrized Gaussian conditional distributions, while that in the low-dimensional space is computed from Student *t*-distributions. The map points are learned by minimizing the Kullback-Leibler (KL) divergence between the probability distributions in the original data space and the embedding space. To map our ISH gene expression data, *x*_*i *_represents the high-dimensional gene expression vector of the *i*th voxel, and *y*_*i *_represents its representation in the low-dimensional space.

In the high-dimensional space, we define the similarity of data point *x*_*j *_to data point *x*_*i *_as the conditional probability *p*_*j*|*i*_, which captures the probability that *x*_*i *_chooses to have *x*_*j *_as its neighbor when neighbors are selected in proportion to their probability density under a Gaussian distribution centered at *x*_*i*_. Formally, the conditional probabilities *p*_*j*|*i *_and *p*_*i*|*j *_are defined as

(1) $$\begin{array}{ll} p_{j|i} & =\frac{\exp\left(-{\left|\left|x_{i}-x_{j}\right|\right|}^{2}/2\sigma_{i}^{2}\right)}{\underset{k\neq i}{\sum }\exp\left(-{\left|\left|x_{k}-x_{i}\right|\right|}^{2}/2\sigma_{i}^{2}\right)},\; \end{array}$$

(2) $$\begin{array}{ll} p_{i|j} & =\frac{\exp\left(-{\left|\left|x_{j}-x_{i}\right|\right|}^{2}/2\sigma_{j}^{2}\right)}{\underset{k\neq j}{\sum }\exp\left(-{\left|\left|x_{k}-x_{j}\right|\right|}^{2}/2\sigma_{j}^{2}\right)},\; \end{array}$$

where $\sigma_{i}^{2}$ denotes the variance of the Gaussian distribution centered at *x*_*i*_. The variance for each data point is tuned separately based on a fixed perplexity specified by the user. The perplexity can be interpreted as a smooth measure of the effective number of neighbors, and it has been shown that the performance of t-SNE is robust to changes in the perplexity [23]. The recommended range for perplexity value is 5 to 50, and the default value of 30 is used in the experiments. Note that *p*_*j*|*i*_≠*p*_*i*|*j*_, and *p*_*i*|*i*_ = 0 for all *i*, since only pairwise similarities are of interests. Then the pairwise similarities in the high-dimensional space are symmetrized as

(3) $$p_{\text{ij}}=\frac{p_{j|i}+p_{i|j}}{2}.$$

The original SNE method employs Gaussian distributions to derive the pairwise similarities in the low-dimensional space. This, however, leads to the crowding problem [23]. To overcome this limitation, the distances in the low-dimensional space are converted into probabilities using a heavy-tailed Student *t*-distribution in t-SNE as

(4) $$q_{\text{ij}}=\frac{{\left(1+{\left|\left|y_{i}-y_{j}\right|\right|}^{2}\right)}^{-1}}{\sum_{k\neq \ell }{\left(1+{\left|\left|y_{k}-y_{\ell }\right|\right|}^{2}\right)}^{-1}}.$$

To learn the map points ${\left\{y_{i}\right\}}_{i=1}^{n}$, t-SNE minimizes the KL divergence between the probability distribution *P* and *Q* in the high-dimensional and low-dimensional spaces as

(5) $$\underset{Q}{\min}\text{KL}(P||Q)=\underset{i}{\sum }\underset{j}{\sum }p_{\text{ij}}\log\frac{p_{\text{ij}}}{q_{\text{ij}}}.$$

Because KL divergence is not symmetric, different types of mismatches contribute differently to the overall cost. Specifically, a large cost will be induced if distant map points are used to represent nearby original data points, while a small cost is incurred if distant original data points are mapped to nearby map points. This indicates that t-SNE is able to preserve the local structure of the high-dimensional data points. It has been shown that the objective function of t-SNE is particularly straightforward to optimize in comparison to the original SNE objective.

The original algorithm in [23] for computing the low-dimensional map points has a time and space complexity of *O*(*n*^2^), where *n* is the number of data points. In [32], a more efficient algorithm, known as the Barnes-Hut-SNE, is developed, and it has O(nlogn) time and *O*(*n*) space complexity. This enables the application of t-SNE to the large-scale Allen Developing Mouse Brain Atlas data. The implementations of t-SNE can be found at http://homepage.tudelft.nl/19j49/t-SNE.html.

### Clustering

To study the relationship between spatial gene expression patterns and classical neuroanatomy in the adult mouse brain, Bohland *et al. *[33] use the Allen Mouse Brain Atlas data [20,34] and apply principal component analysis (PCA) to reduce the data dimensionality before the *k*-means algorithm is used to cluster the brain voxels into groups. To visualize the spatial gene expression patterns, they also map the high-dimensional gene expression data to 3-D space using PCA and visualize the data using scatter plots.

Following [33], we apply the *k*-means clustering algorithms to group brain voxels into clusters based on the gene expression data in both the original high-dimensional space and the dimensionality-reduced space. Since the results of the *k*-means algorithm depend on the initial cluster centers that are randomly selected, we repeat this algorithm 10 times and use the results with the smallest within-cluster sum of squares error. The number of clusters in *k*-means is set to be equal to the number of brain structures at each particular ontology level. We reduce the high-dimensional gene expression data to 2-D and 10-D spaces using t-SNE and PCA and then apply the *k*-means algorithm to cluster the voxels based on these low-dimensional representations. We then quantitatively compare the consistency between voxel clusters and the neuroanatomy at multiple levels in the Reference Atlas developmental ontology.

We employ four performance measures, including the normalized mutual information (NMI), adjusted rand index (ARI), purity, and S-index, to evaluate the consistency between clustering results and developmental neuroanatomy. The first three measures have been commonly used in the clustering community as external criteria for evaluating clustering results [35], and the ARI and S-index have been used for comparing different brain parcellation schemes [12]. We treat the voxel annotations as their class labels and compare them with the clustering results. In computing purity, each cluster is assigned to the most frequent class in the cluster, and then the final measure is the proportion of correctly assigned samples. One disadvantage of purity is that it cannot trade off the quality of the clustering against the number of clusters [35]. This limitation can be overcome by the NMI, which measures the amount of (normalized) information by which our knowledge about the classes increases when we are given the clustering results. The ARI computes the normalized fraction of all possible pairs of voxels that (1) have the same class label and are assigned to the same cluster or (2) have different class labels and are assigned to different clusters. The S-index was specifically designed to compare different brain parcellations, and it “penalizes” class-to-cluster relationships that are overlapping, but that are not pure subset relationships [12]. Different measures capture different aspects of class-to-cluster consistency, and thus the trend of performance by different measures might not always be the same.

## Results and discussion

We retrieve the ISH expression energy grid data, the Reference Atlas ontology and annotation data for all seven developmental ages from the Allen Brain Atlas API [36]. We remove voxels in the spinal cord for all developmental ages, as our primary goal is to study the brain gene expression and anatomy. We also remove voxels that are annotated to a level less than Level 5, since we are interested in studying the relationship between the spatial gene expression patterns and the transversal and longitudinal grid-like domains that correspond to Level 5 annotations in the Reference Atlas ontology. In the current release of the data (October, 2012), most of the annotations have been worked down to levels between 5 and 8; hence only a small number of voxels were removed in this step. After this step, all voxels are annotated with structures at levels between 5 and 12. To study the developing mouse brain anatomy at multiple levels of granularity, we propagate the annotation of each voxel up to Level 5, Level 3, and Level 1, resulting in three annotated structures for each voxel that correspond to ancestor-child relations in the Reference Atlas ontology. The statistics of the data sets that are used in this work are shown in Table 2. The input data to the t-SNE method for each developmental stage is a data matrix of size *n*×*d*, where *n* is the number of voxels, and *d* is the number of genes.

**Table 2:** Statistics of the Allen Developing Mouse Brain Atlas data sets that are used in this work

**Age**	**E11.5**	**E13.5**	**E15.5**	**E18.5**	**P4**	**P14**	**P28**
# of genes	1948	1948	1930	1946	1918	1906	1944
# of voxels	5021	9541	11694	11928	21682	24313	27991

The data sets span seven developmental ages, and the number of genes and the number of voxels are reported.

### Data visualization at multiple ontology levels

**Figure 4:**
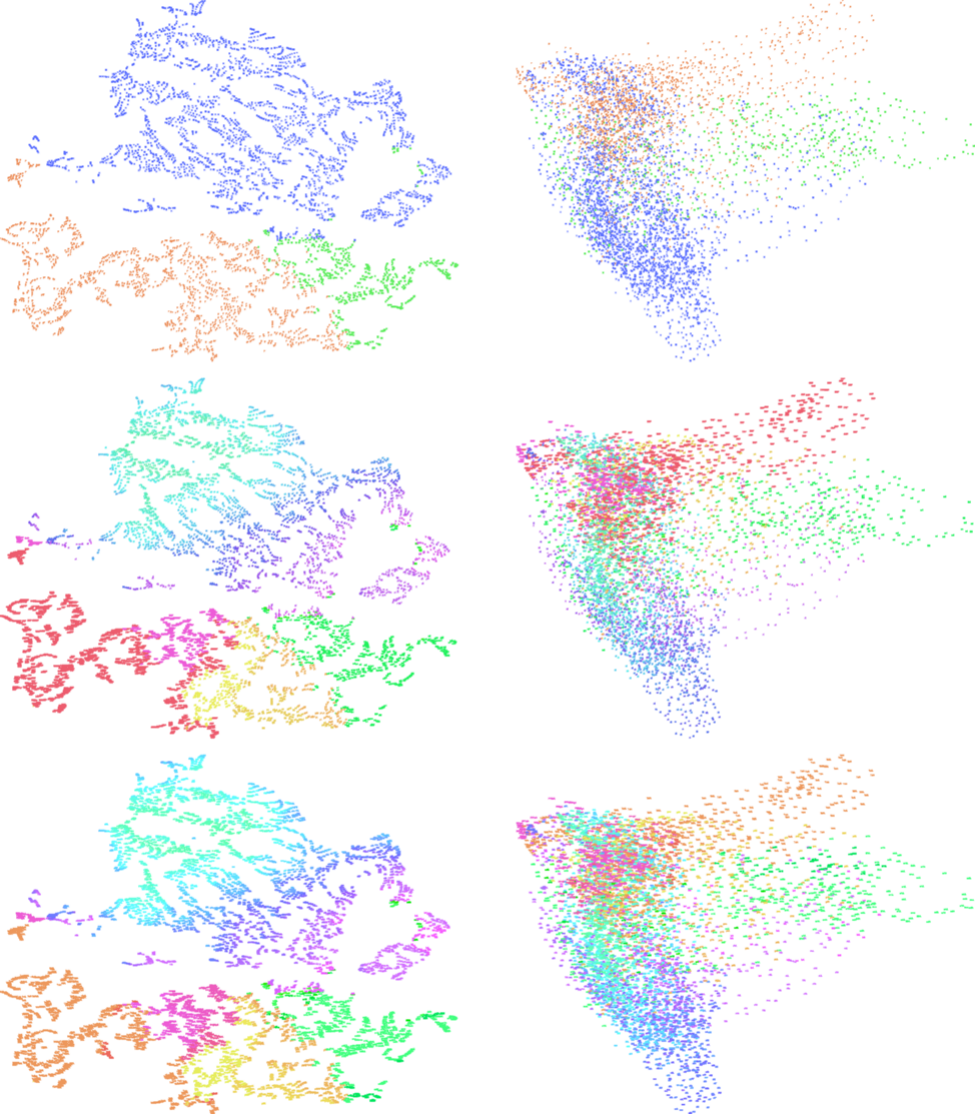
**Visualization of the Allen Developing Mouse Brain Atlas data for age E11.5 after projecting to 2-D space using t-SNE (left column) and PCA (right column) at multiple levels of the ontology.** The three rows correspond to Levels 1, 3, and 5. Each point corresponds to a brain voxel, which is displayed using the structure abbreviation and color of its Reference Atlas annotation. The structure abbreviations can be seen by zooming into each figure, and the structure name can be found in Figure 3.

**Figure 5:**
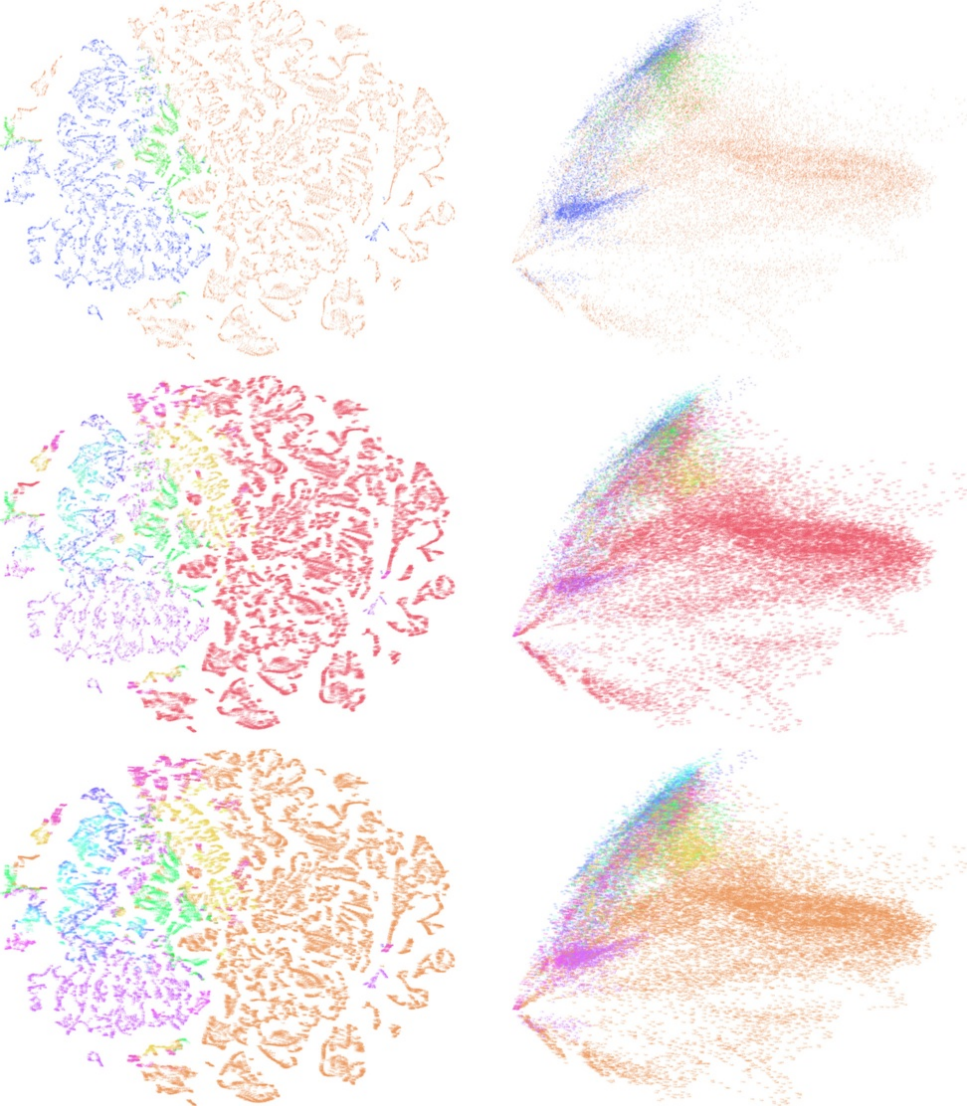
**Visualization of the Allen Developing Mouse Brain Atlas data for age P28 after projecting to 2-D space using t-SNE (left column) and PCA (right column) at multiple levels of the ontology.** The three rows correspond to Levels 1, 3, and 5. Each point corresponds to a brain voxel, which is displayed using the structure abbreviation and color of its Reference Atlas annotation. Results for other ages are shown in the Additional file 1.

To visually explore the relationship between spatial gene expression patterns and brain neuroanatomy, we project the high-dimensional, voxel-level gene expression vectors onto 2-D space using t-SNE and PCA. In PCA, the data matrices are centered by subtracting the mean. To investigate this relationship at multiple levels of the ontology, we display each projected data point using its Level 1, Level 3, and Level 5 annotations, where the structure abbreviation is used as the marker that is color-coded according to its Reference Atlas ontology color. The full names of structures can be found in Figure 3. We show the results generated by t-SNE and PCA using Level 1, Level 3, and Level 5 annotations in Figures 4 and 5 for ages E11.5 and P28, respectively. The complete set of visualization results for all other ages are included in the Additional file 1.

We observe that t-SNE is better at visualizing the high-dimensional gene expression data than PCA. Specifically, we can observe that, at all developmental ages, the three major brain structures at Level 1 (forebrain, midbrain, and hindbrain) are very well separated. The results by t-SNE preserve the brain anatomy more faithfully than those by PCA at this level. The second rows of Figures 4 and 5 show the results by t-SNE and PCA displayed using the Level 3 annotations, which identify the major transversal segments. We can observe that both the global and local brain structures at this level are largely preserved in the dimensionality-reduced gene expression data space. The third rows of Figures 4 and 5 show the scatter plots of reduced data displayed using the Level 5 annotations, which identify the four longitudinal zones in addition to the transversal segments. We can observe that within each of the transversal segments, voxels belong to the same longitudinal zones are usually placed close to each other. However, voxels in the same longitudinal zone but belong to different transversal segments are not necessarily placed at nearby locations.

We can observe from Figures 4 and 5 that t-SNE is able to map high-dimensional data to 2-D space in which the neuroanatomy can be largely recovered. For example, in Figures 4 and 5 the overall organization of the three brain structures at Level 1 are largely preserved, where the midbrain voxels are placed between the forebrain and hindbrain voxels. These results indicate that t-SNE is able to preserve both the local and the global structures of the data simultaneously. In addition, the shapes of the structures are also preserved to some extent. For example, it is known that the midbrain is a wedge-shaped structure due to the sharp flexion of the neuraxis in this region [37]. We can see from Figures 4 and 5 that this is largely preserved in most plots. This is especially clear from plot for the developmental age E11.5. This is presumably due to the much larger number of voxels in late ages (Table 2), which prevent some global structures from being fully incorporated.

At Level 3 shown in Figures 4 and 5, the transversal segment structures are also largely preserved. In particular, p1 voxels are almost always close to the midbrain voxels, while p3 voxels are usually on the secondary prosencephalon side. m1 voxels are mostly placed closely with p1 voxels, while m2 voxels are nearby with hindbrain voxels. In the hindbrain, prepontine hindbrain voxels (including is, r1, and r2) are mostly close to midbrain voxels; medullary hindbrain voxels (including r7, r8, r9, r10, and r11) are placed on the far side; pontine hindbrain (r3 and r4) and pontomedullary hindbrain (r5 and r6) voxels are somewhere in between. We also observe that the global brain structures are less well preserved at late developmental ages. This might be due to the increasingly larger number of brain voxels at late ages, which makes it increasingly difficult to preserve both the global and the local structures. In this case, t-SNE tends to focus more on retaining the local structure due to the asymmetric nature of the KL divergence.

### Clustering and comparison with neuroanatomy

Since our visual explorations have shown that the brain anatomy is largely preserved in the dimensionality-reduced space, we expect that grouping of voxels into clusters based on the low-dimensional representations might lead to voxel clusters that are more consistent with neuroanatomy than those obtained from the original high-dimensional representations. We use t-SNE and PCA to reduce the data to 2-D and 10-D spaces and then apply the *k*-means clustering algorithm to group the low-dimensional representations. We employ four performance measures to evaluate the consistency between clustering results and neuroanatomy. The results at developmental ontology levels 1, 3, and 5, respectively, are reported in Tables 3, 4, and 5.

**Table 3:** Comparison of clustering results with the Reference Atlas annotations at developmental ontology Level 1

**Measures**	**Clustering**	**E11.5**	**E13.5**	**E15.5**	**E18.5**	**P4**	**P14**	**P28**	**Average**
	*k*-means	0.2045	0.1200	0.1622	0.1969	0.1257	0.1395	0.1871	0.1623
NMI	PCA_2_	0.2076	0.1047	0.1651	0.1959	0.1252	0.1436	0.1931	0.1622
	PCA_10_	0.2034	0.1181	0.1602	0.1956	0.1257	0.1399	0.1866	0.1614
	t-SNE_2_	0.4781	0.6038	0.5022	0.1927	0.3186	0.3387	0.3833	0.4025
	t-SNE_10_	0.5650	0.7770	0.5385	0.2417	0.3294	0.4090	0.3206	0.4544
	*k*-means	0.2874	0.1271	0.1457	0.2644	0.2136	0.2067	0.1687	0.2019
S-index	PCA_2_	0.2891	0.0959	0.2114	0.4320	0.1337	0.2082	0.1170	0.2125
	PCA_10_	0.2840	0.1482	0.1468	0.2628	0.2123	0.1468	0.1537	0.1935
	t-SNE_2_	0.4153	0.6151	0.5265	0.1138	0.5025	0.3578	0.2957	0.4038
	t-SNE_10_	0.5008	0.8106	0.3719	0.1171	0.7396	0.6931	0.4038	0.5196
	*k*-means	0.2044	0.0944	0.0976	0.1423	0.0303	0.0251	0.0950	0.0985
ARI	PCA_2_	0.2210	0.1007	0.0949	0.1411	0.0308	0.0330	0.1012	0.1032
	PCA_10_	0.2057	0.0934	0.0959	0.1415	0.0313	0.0257	0.0950	0.0984
	t-SNE_2_	0.4601	0.6197	0.4210	0.1442	0.2124	0.2611	0.2961	0.3450
	t-SNE_10_	0.5876	0.8101	0.4539	0.2282	0.1119	0.3427	0.1643	0.3855
	*k*-means	0.6359	0.6124	0.6668	0.6885	0.7128	0.7038	0.7204	0.6772
Purity	PCA_2_	0.6469	0.6069	0.6639	0.6877	0.7128	0.7038	0.7218	0.6777
	PCA_10_	0.6365	0.6117	0.6654	0.6878	0.7128	0.7038	0.7191	0.6767
	t-SNE_2_	0.7554	0.8342	0.8304	0.6743	0.8084	0.8446	0.8567	0.8006
	t-SNE_10_	0.8054	0.9216	0.8037	0.6571	0.7503	0.8748	0.7881	0.8001

“*k*-means” denotes applications of the *k*-means to the original high-dimensional gene expression data; “PCA_2_” and “PCA_10_” denote applications of *k*-means to PCA reduced data, where the first 2 and 10 principal components are used, respectively; “t-SNE_2_” and “t-SNE_10_” denote applications of *k*-means to data reduced to 2 and 10 dimensions, respectively, by t-SNE. Four different measures, including normalized mutual information (NMI), S-index, adjusted rand index (ARI), and purity, are used to compare the clustering results to the reference atlas annotations.

**Table 4:** Comparison of clustering results with the Reference Atlas annotations at developmental ontology Level 3

**Measures**	**Clustering**	**E11.5**	**E13.5**	**E15.5**	**E18.5**	**P4**	**P14**	**P28**	**Average**
	*k*-means	0.4173	0.3754	0.3882	0.3620	0.3633	0.3480	0.3455	0.3714
NMI	PCA_2_	0.2545	0.1508	0.1777	0.2344	0.2101	0.2631	0.2718	0.2232
	PCA_10_	0.3831	0.3305	0.3310	0.3045	0.3315	0.3094	0.3247	0.3307
	t-SNE_2_	0.5337	0.4947	0.5097	0.3977	0.3556	0.3481	0.3637	0.4290
	t-SNE_10_	0.5469	0.5202	0.5065	0.4382	0.4420	0.3484	0.3947	0.4567
	*k*-means	0.5820	0.5751	0.5605	0.5815	0.5889	0.6838	0.5839	0.5937
S-index	PCA_2_	0.6524	0.6869	0.6206	0.6413	0.6399	0.6790	0.6283	0.6498
	PCA_10_	0.6065	0.6547	0.6270	0.6050	0.6278	0.6982	0.6113	0.6329
	t-SNE_2_	0.4729	0.5132	0.5387	0.5256	0.5889	0.6736	0.6383	0.5645
	t-SNE_10_	0.4724	0.4941	0.5736	0.5968	0.5566	0.6653	0.5673	0.5609
	*k*-means	0.1756	0.1516	0.1341	0.1242	0.0609	0.0571	0.0634	0.1096
ARI	PCA_2_	0.0849	0.0235	0.0066	0.0327	0.0190	0.0386	0.0499	0.0364
	PCA_10_	0.1839	0.1175	0.0980	0.0700	0.0581	0.0388	0.0531	0.0885
	t-SNE_2_	0.2654	0.1967	0.1670	0.1021	0.0823	0.0779	0.0788	0.1386
	t-SNE_10_	0.2699	0.2123	0.1521	0.1195	0.0902	0.0847	0.0965	0.1464
	*k*-means	0.3854	0.5233	0.6028	0.6135	0.6853	0.7253	0.7090	0.6064
Purity	PCA_2_	0.3127	0.3825	0.4584	0.5190	0.6056	0.6594	0.6473	0.5121
	PCA_10_	0.3918	0.4835	0.5596	0.5560	0.6708	0.6856	0.6892	0.5766
	t-SNE_2_	0.4774	0.5824	0.6772	0.6082	0.6795	0.7072	0.7121	0.6349
	t-SNE_10_	0.4895	0.6081	0.6473	0.6392	0.7305	0.7306	0.7342	0.6542

See the footnote of Table 3 for detailed explanations.

**Table 5:** Comparison of clustering results with the Reference Atlas annotations at developmental ontology Level 5

**Measures**	**Clustering**	**E11.5**	**E13.5**	**E15.5**	**E18.5**	**P4**	**P14**	**P28**	**Average**
	*k*-means	0.5932	0.5264	0.5009	0.4493	0.4210	0.4165	0.3885	0.4708
NMI	PCA_2_	0.3429	0.2243	0.2269	0.2781	0.2390	0.2931	0.2877	0.2703
	PCA_10_	0.5354	0.4481	0.4299	0.4011	0.3815	0.3796	0.3720	0.4211
	t-SNE_2_	0.6267	0.5593	0.5331	0.4932	0.4222	0.4100	0.4228	0.4953
	t-SNE_10_	0.6321	0.5736	0.5279	0.5062	0.4600	0.4089	0.4564	0.5093
	*k*-means	0.5822	0.6408	0.6675	0.6727	0.7024	0.7380	0.7234	0.6753
S-index	PCA_2_	0.8000	0.8380	0.8329	0.8017	0.8161	0.7819	0.8128	0.8119
	PCA_10_	0.6712	0.7064	0.7288	0.7258	0.7398	0.7506	0.7392	0.7231
	t-SNE_2_	0.5562	0.6257	0.6670	0.6714	0.6901	0.7441	0.7318	0.6695
	t-SNE_10_	0.5603	0.6096	0.6515	0.6685	0.6997	0.7077	0.7112	0.6584
	*k*-means	0.1380	0.0839	0.0634	0.0467	0.0331	0.0318	0.0272	0.0606
ARI	PCA_2_	0.0428	0.0135	0.0108	0.0211	0.0124	0.0169	0.0214	0.0199
	PCA_10_	0.1132	0.0660	0.0488	0.0384	0.0264	0.0238	0.0252	0.0488
	t-SNE_2_	0.1698	0.0946	0.0675	0.0631	0.0337	0.0316	0.0306	0.0701
	t-SNE_10_	0.1765	0.1087	0.0618	0.0709	0.0448	0.0304	0.0444	0.0768
	*k*-means	0.4961	0.6097	0.6437	0.6192	0.7062	0.7663	0.7272	0.6526
Purity	PCA_2_	0.2842	0.3640	0.4556	0.5260	0.5929	0.6666	0.6545	0.5063
	PCA_10_	0.4314	0.5330	0.5947	0.6006	0.6882	0.7430	0.7199	0.6158
	t-SNE_2_	0.5346	0.6254	0.6729	0.6515	0.6943	0.7533	0.7465	0.6684
	t-SNE_10_	0.5383	0.6456	0.6711	0.6609	0.7309	0.7480	0.7623	0.6796

See the footnote of Table 3 for detailed explanations.

We can observe from Table 3 that the results from low-dimensional representations computed by t-SNE are much more consistent with neuroanatomy than those from the original representations at Level 1. On average, the performance measured by NMI and S-index has been more than doubled, and that by adjusted rand index has been increased from 0.0985 to 0.3855. On the other hand, the results from PCA-reduced data are similar to those by the original data. This is consistent with the visualization results that PCA-reduced data fail to separate voxels from different brain structures clearly at this level. We also observe that the results of PCA are similar to those by the original data for measures NMI, ARI, and purity. For S-index, these two sets of results are not similar. This might indicate that S-index measures class-to-cluster consistency in a different way than other measures. As has been mentioned in Section “Clustering”, S-index penalizes class-to-cluster relationships that are overlapping, but that are not pure subset relationships [12]. The other three measures are not specifically designed to capture such relationship.

At Levels 3 and 5, we can observe from Tables 4 and 5 that, on average, the clustering results based on the t-SNE reduced data are more consistent with the neuroanatomy than those by the original data. In addition, the t-SNE results are more consistent with the neuroanatomy than those by PCA for measures NMI, ARI, and purity. The PCA-reduced data give better performance than the original and the t-SNE reduced data for measure S-index. This again indicates that S-index measures consistency in a different way compared with the other three measures. We can conclude from the above results that, although t-SNE gives better visualization results than PCA at all levels, the clustering results based on PCA-reduced data could yield higher consistency with the neuroanatomy than those based on t-SNE for certain measure. These results are consistent with the results reported in [33].

### Dimensionality reduction by t-SNE and PCA

We observe that t-SNE gives the best results in terms of preserving both the local and the global structures in the high-dimensional gene expression space in comparison with PCA. We also observe that when the data sets are very large, such as those in late developmental ages of the Allen Developing Mouse Brain Atlas, preserving both the local and the global structures might be very hard or even impossible. In these cases, t-SNE tries to preserve local structures at the price of losing some global structures. This tradeoff is achieved by giving different costs to different types of errors in computing the mapping. In particular, because KL divergence is not symmetric, different types of mismatches contribute differently to the overall cost. A large cost will be induced if distant map points are used to represent nearby original data points. This large cost will ensure that the local structures are faithfully preserved. In contrast, a relatively small cost is incurred if distant original data points are mapped to nearby map points. Hence, a small cost will be incurred if the global structures are not preserved accurately. This asymmetric property makes t-SNE especially useful in reducing and visualizing large-scale brain data sets in comparison to other traditional techniques, which preserve either the global or the local structures.

### Longitudinal zones versus transversal segments

In developmental neuroanatomy, two primary models have been proposed to explain the neural plate and tube regionalization based on gene expression and morphological information [13]. These are the topographic “columnar” model [38], and the topological “segmental” model known as the “prosomeric model” [24-26,39]. Recent experimental data have shown that the prosomeric model is more consistent with morphological and molecular evidences. This leads to the adoption of this model in the Allen Developing Mouse Brain Reference Atlas. The columnar model mainly focuses on dividing the neural plate and tube along the longitudinal dimension, while the segmental model favors division into transversal domains. In the prosomeric model (Figure 1), the developing nervous system is divided into a grid-like pattern of longitudinal and transversal histogenetic domains. Along the longitudinal axis, four zones, known as the floor plate, basal plate, alar plate, and roof plate, are specified by DV patterning mechanisms. Along the transversal axis, the AP patterning signals subdivide the brain wall into a constant set of segments known as neuromeres.

**Figure 6:**
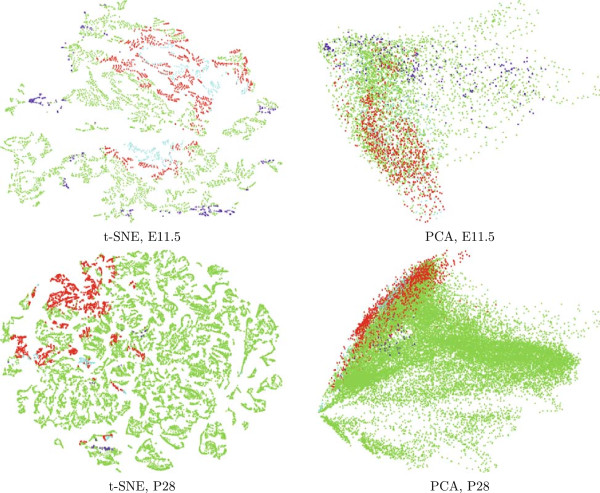
**Visualization of the Allen Developing Mouse Brain Atlas data for ages E11.5 and P28 after projecting to 2-D space using t-SNE and PCA.** Each point corresponds to a brain voxel, which is displayed according to the longitudinal zones (F=floor, B=basal, A=alar, R=roof) it belongs to. Results for other ages are shown in the Additional file 1.

To provide in-depth visual exploration of the genetic neuroanatomy along the longitudinal and transversal dimensions, we display in Figure 6 the E11.5 and P28 data sets according to the longitudinal zone that each voxel belongs to. These results can be compared with the Level 3 visualizations in Figures 4 and 5, which displays the reduced data according to the transversal segment that each voxel belongs to. We can observe from the t-SNE results that voxels from the same longitudinal zones do not form clear clusters in comparison to the clustering patterns along the transversal dimension. In general, voxels belongs to the alar plate and basal plate form clear clusters, while those in the roof plate and floor plate tend to be widely distributed. However, we can observe that voxels in the roof and alar plates are usually close to each other, and those in the basal and floor plates tend to form clusters. This shows that our computational results are more consistent with the segmental model, which is also supported by recent experimental evidences.

### Manifold structures in developmental gene expression

We have observed that clustering of the low-dimension representations generated by t-SNE leads to more consistent results with neuroanatomy than those by the original and the PCA-reduced representations. This might indicate that the original gene expression data lie on a low-dimensional manifold in the high-dimensional space. In addition, a general trend that we have observed in comparing the clustering results with neuroanatomy is that clustering using the low-dimensional representations gives very significant performance improvement at Level 1 in comparison to those by the original and the PCA-reduced representations. This improvement decreases as we move to Level 3 and Level 5. Such trend is consistent with our hypothesis that the original gene expression data lie on a manifold in the high-dimensional space, because the Level 1 structures are simpler and thus are easier to capture by low-dimensional representations than those at Level 3 and Level 5. Hence, embedding of the simple manifold into low-dimensional space facilitates the faithful characterization of the underlying structures. On the other hand, reducing relatively complex manifold structures to low-dimensional space might not lead to better representations.

## Conclusions

We employ global computational analysis to study the relationship between gene expression patterns and neuroanatomy in the developing mouse brain. To enable visual explorations, we map the high-dimensional ISH gene expression data to low-dimensional space by preserving both the local and the global structures. This unsupervised, data-driven mapping of spatial gene expression data leads to low-dimensional representations that can be easily visualized. Our results show that the developmental neuroanatomy is largely preserved in the low-dimensional gene expression data space. To provide quantitative results, we cluster both the original high-dimensional data and the low-dimensional mapped data and compare the results with the developmental neuroanatomy. Our results show that the clusters in the low-dimensional space are more consistent with developmental neuroanatomy than those in the high-dimensional space.

In this work, the data set at each developmental age is analyzed separately. Since development is a continuous process, it would be interesting to map and cluster the data by incorporating temporal smoothness constraints [40,41]. We will explore time-varying dimensionality reduction and clustering algorithms in the future. Our results have shown that, although majority of the voxels are mapped to locations that are consistent with their anatomical annotations, there do exist some exceptions. We will investigate these cases in the future.

## Competing interests

The author declares no competing interests.

## Supplementary Material

Additional file 1 **The additional file contains the complete set of visualization results for developmental ages not shown in the main texts**. Click here for file
