# Supplementary material for: Computational genetic neuroanatomy of the developing mouse brain: dimensionality reduction, visualization, and clustering
Source: BMC Bioinformatics. 2013 Jul 11;14:222. doi: 10.1186/1471-2105-14-222 (PMC3750329; doi:10.1186/1471-2105-14-222)
Supplement: Additional file 1 — The additional file contains the complete set of visualization results for developmental ages not shown in the main texts. [file 1471-2105-14-222-S1.pdf]

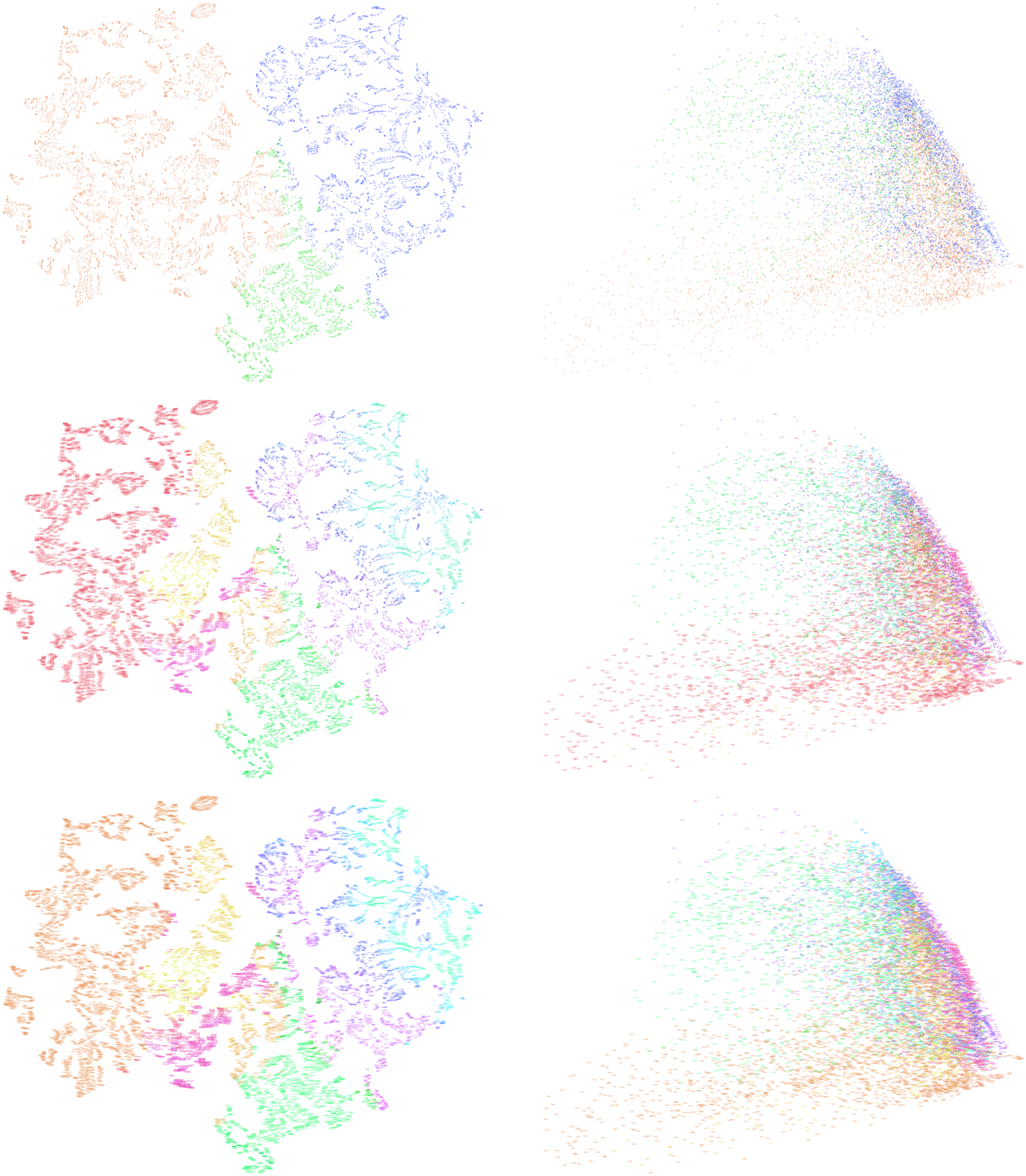

Figure 1: Visualization of the Allen Developing Mouse Brain Atlas data for age E13.5 after projecting to 2-D space using t-SNE (left column) and PCA (right column) at multiple levels of the ontology. The three rows correspond to Levels 1, 3, and 5. Each point corresponds to a brain voxel, which is displayed using the structure abbreviation and color of its Reference Atlas annotation.

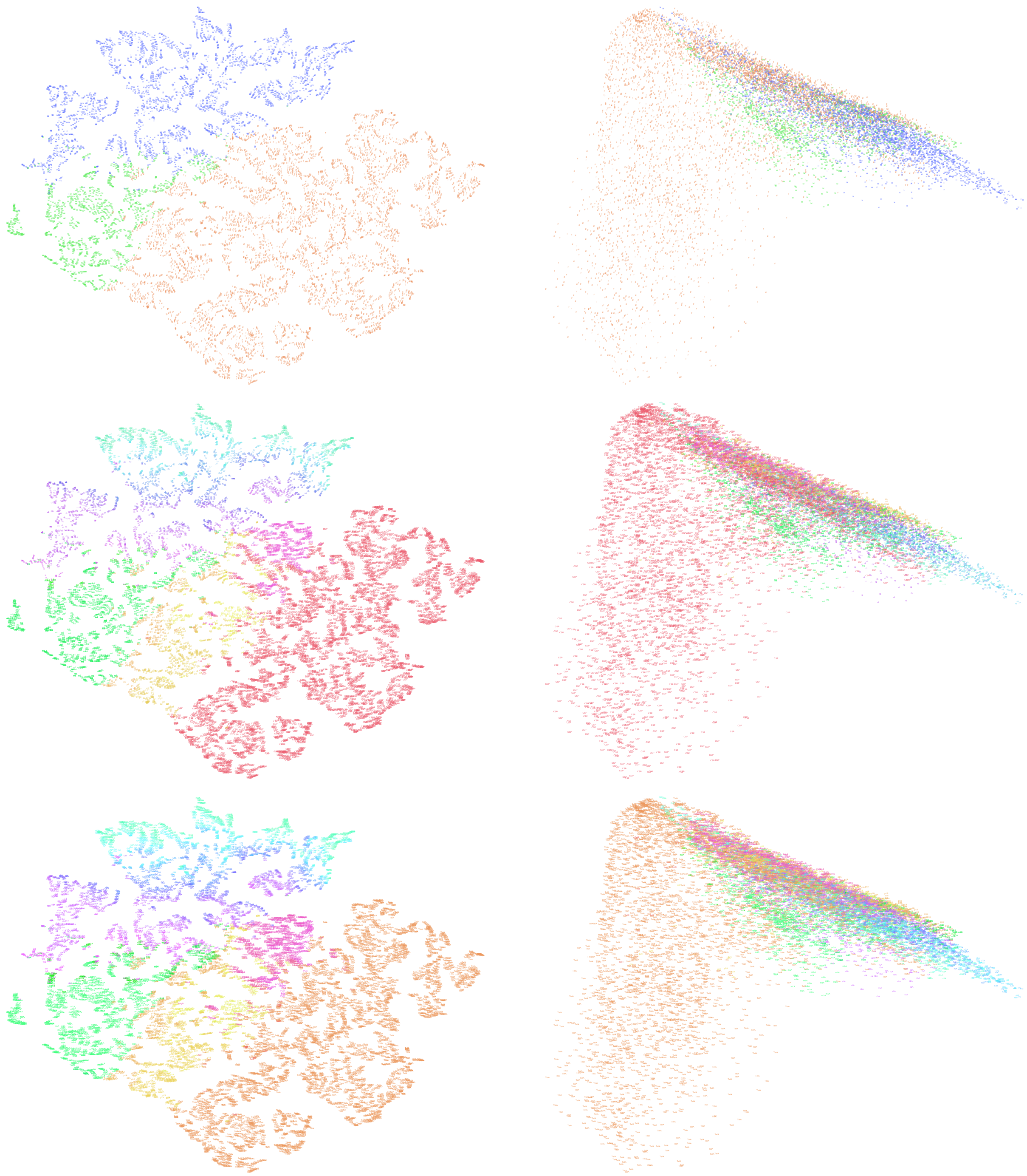

Figure 2: Visualization of the Allen Developing Mouse Brain Atlas data for age E15.5 after projecting to 2-D space using t-SNE (left column) and PCA (right column) at multiple levels of the ontology. The three rows correspond to Levels 1, 3, and 5. Each point corresponds to a brain voxel, which is displayed using the structure abbreviation and color of its Reference Atlas annotation.

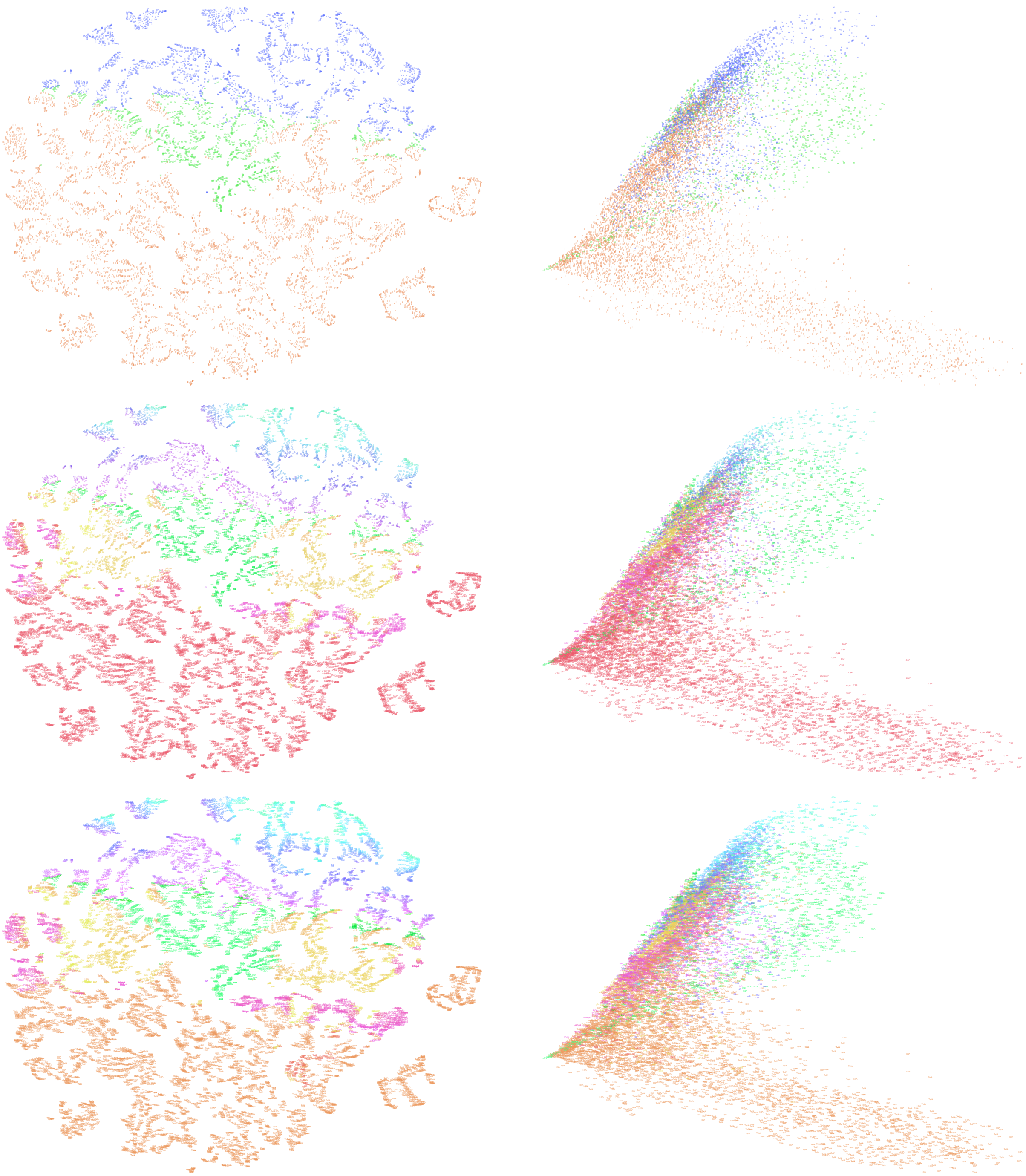

Figure 3: Visualization of the Allen Developing Mouse Brain Atlas data for age E18.5 after projecting to 2-D space using t-SNE (left column) and PCA (right column) at multiple levels of the ontology. The three rows correspond to Levels 1, 3, and 5. Each point corresponds to a brain voxel, which is displayed using the structure abbreviation and color of its Reference Atlas annotation.

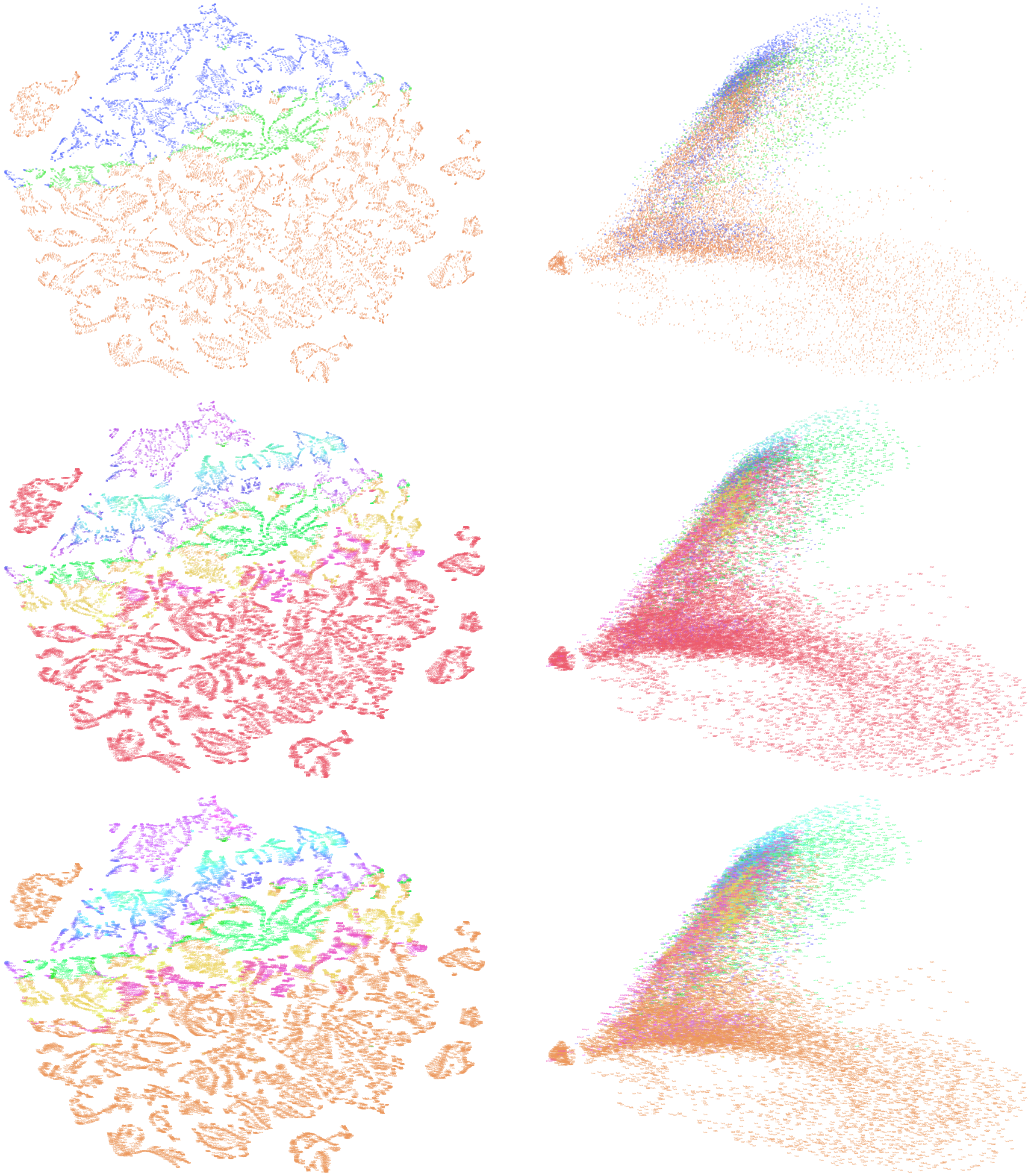

Figure 4: Visualization of the Allen Developing Mouse Brain Atlas data for age P4 after projecting to 2-D space using t-SNE (left column) and PCA (right column) at multiple levels of the ontology. The three rows correspond to Levels 1, 3, and 5. Each point corresponds to a brain voxel, which is displayed using the structure abbreviation and color of its Reference Atlas annotation.

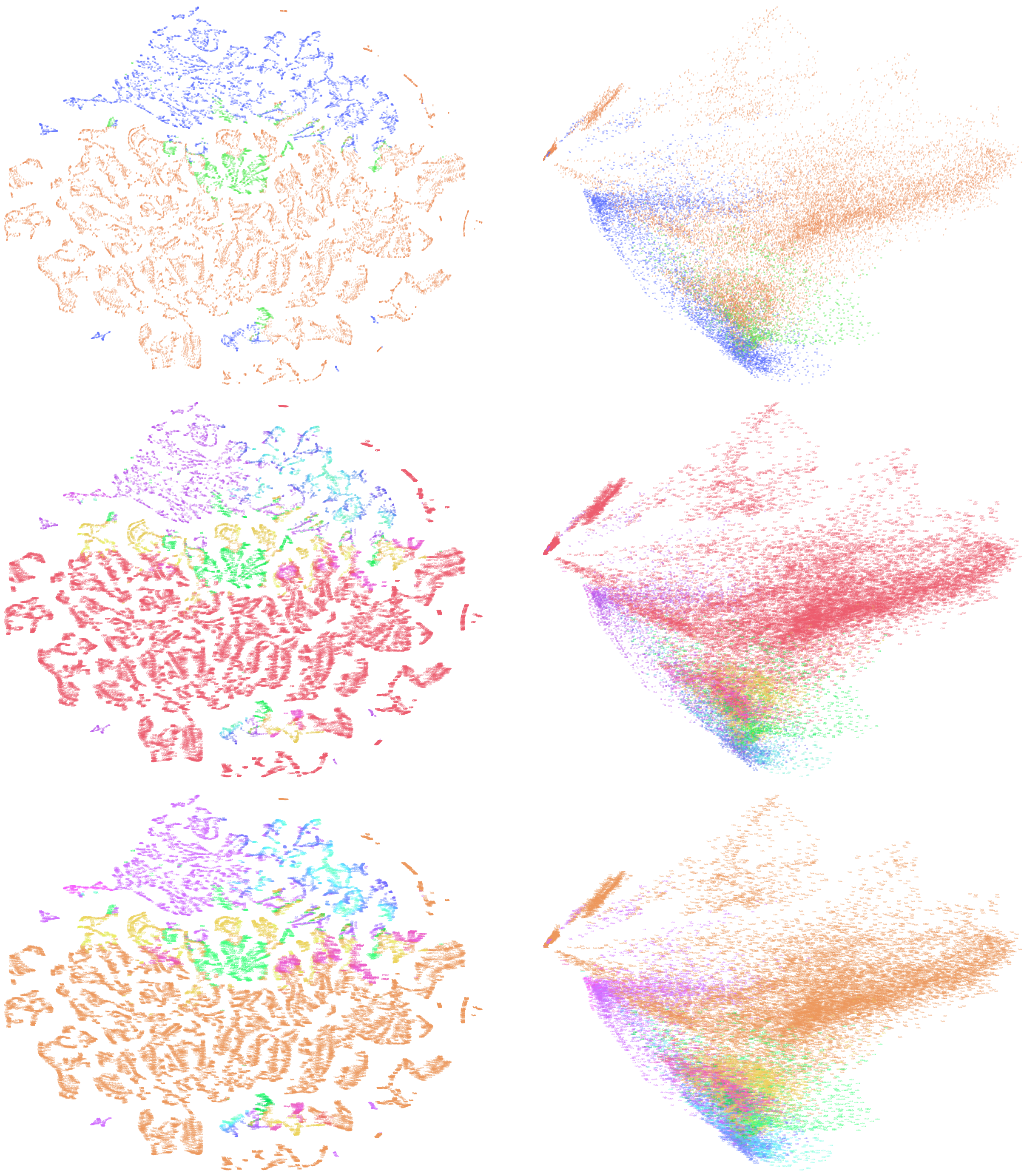

Figure 5: Visualization of the Allen Developing Mouse Brain Atlas data for age P14 after projecting to 2-D space using t-SNE (left column) and PCA (right column) at multiple levels of the ontology. The three rows correspond to Levels 1, 3, and 5. Each point corresponds to a brain voxel, which is displayed using the structure abbreviation and color of its Reference Atlas annotation.

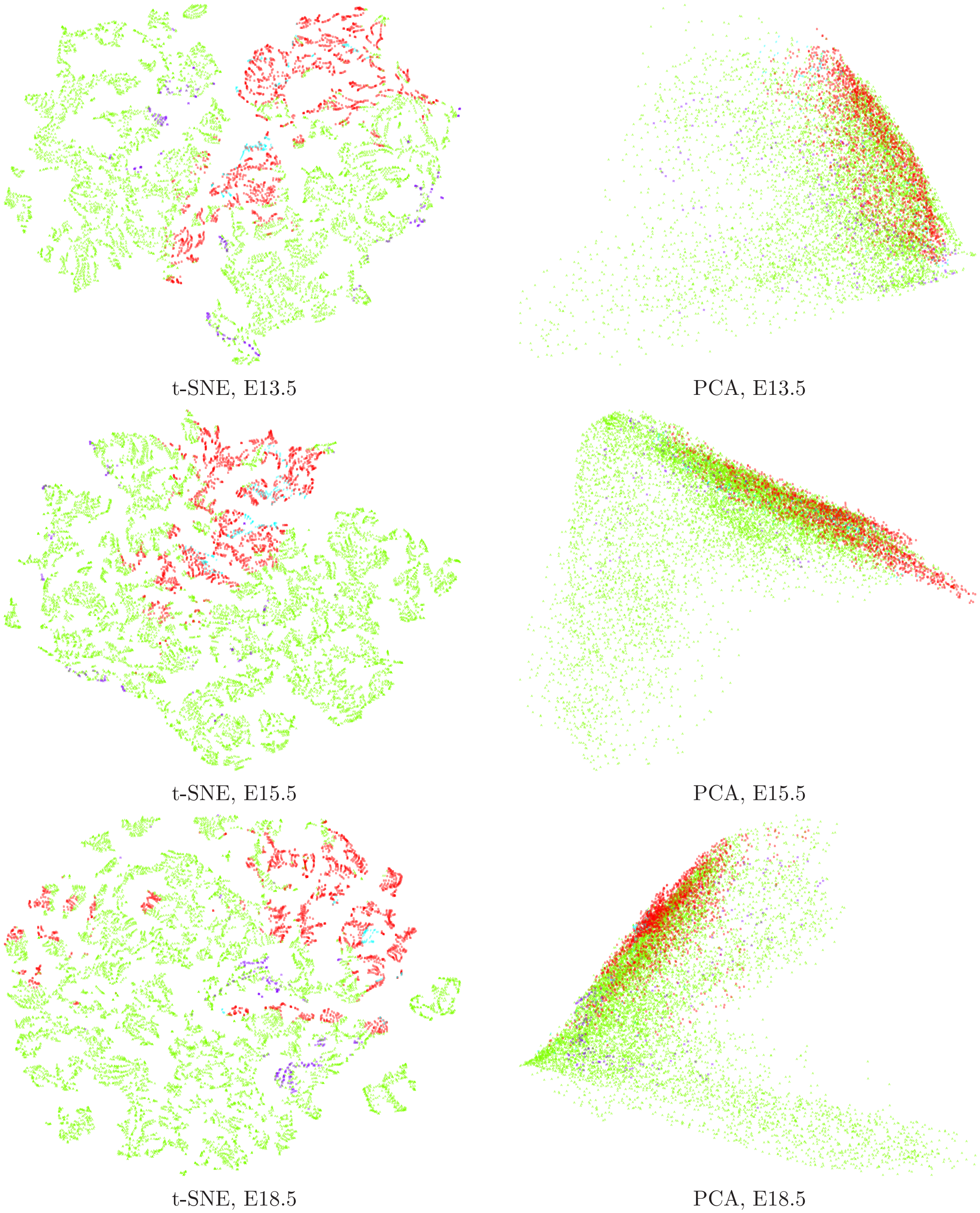

Figure 6: Visualization of the Allen Developing Mouse Brain Atlas data after projecting to 2-D space using t-SNE and PCA. Each point corresponds to a brain voxel, which is displayed according to the longitudinal zones (F=floor, B=basal, A=alar, R=roof) it belongs to.

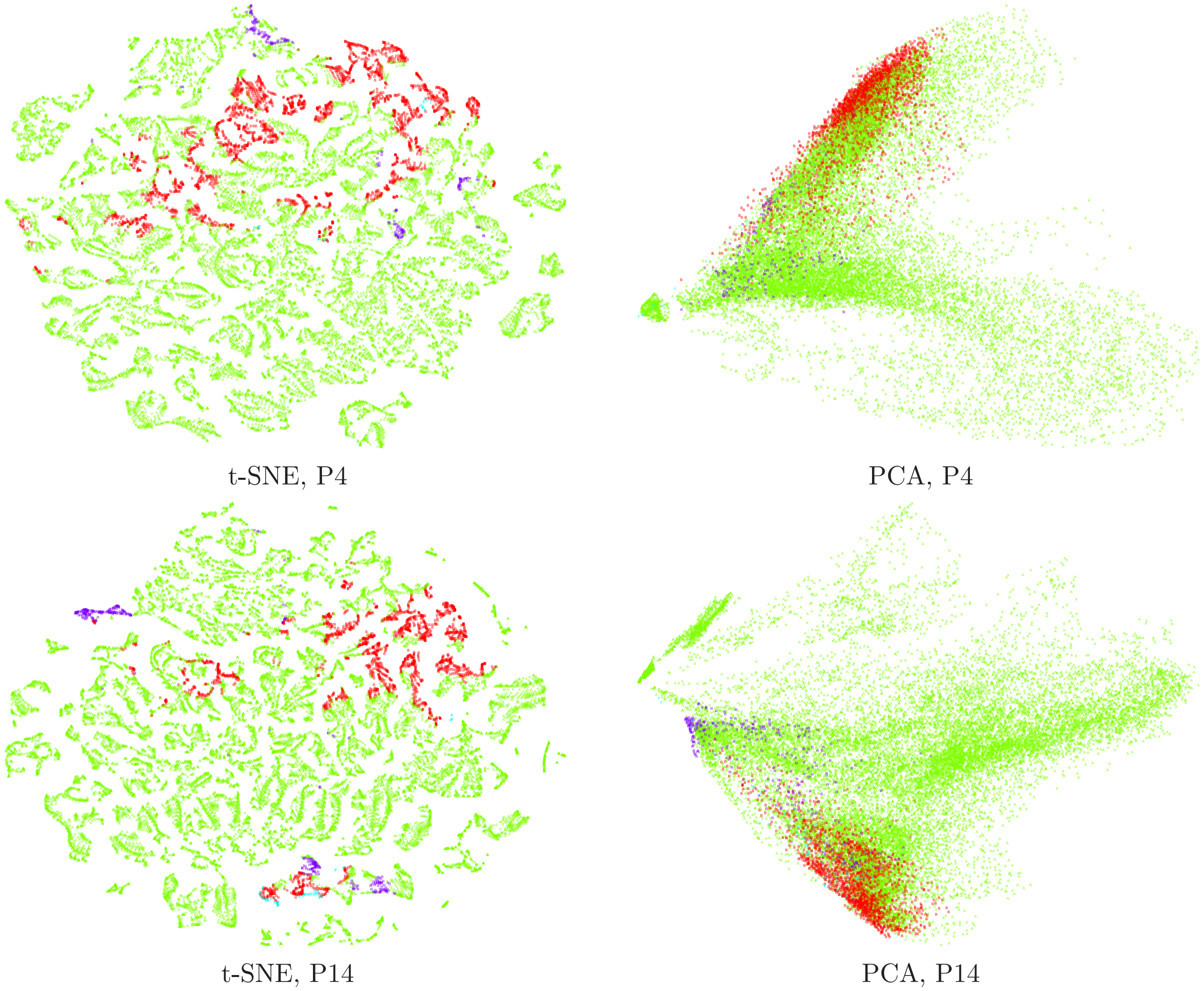

Figure 7: Visualization of the Allen Developing Mouse Brain Atlas data after projecting to 2-D space using t-SNE and PCA. Each point corresponds to a brain voxel, which is displayed according to the longitudinal zones (F=floor, B=basal, A=alar, R=roof) it belongs to.
